# Perceived COVID-19 Vaccine Pressure in the Caribbean: Exploring a New Stressor–Strain Phenomenon in the Pandemic

**DOI:** 10.3390/vaccines10020238

**Published:** 2022-02-03

**Authors:** Dwayne Devonish, Teixiera Dulal-Arthur

**Affiliations:** Cave Hill Campus, University of the West Indies, Cave Hill, Saint Michael BB22026, Barbados; teixieradulalarthur@gmail.com

**Keywords:** perceived COVID-19 vaccine pressure, mental health, psychological strain, Barbados, vaccine, stress

## Abstract

This research introduced the new construct of ‘perceived COVID-19 vaccine pressure’ (i.e., the psychological strain associated with societal demands on vaccine taking) and examined the initial psychometric properties of a newly proposed measure. The study surveyed 411 Barbadian respondents to examine their level of perceived COVID-19 vaccine pressure using an online survey modality. The results revealed strong and robust psychometric properties for the scale and its unidimensionality. Younger and employed respondents as well as those working in the tourism and hospitality and government (public) sectors experienced the greatest internalised vaccine pressure relative to other respondent groups. Only initial/preliminary evidence of the scale’s validity and reliability was revealed by this cross-sectional study. A follow-up study (using CFA on a new sample) is needed to provide stronger evidence for its validity and reliability. Understanding the dynamics of perceived or internalised vaccine pressure might help explain the positive or negative effects of societal pressure and its implications for vaccine hesitancy and other vaccine-related attitudes and behaviours. The study is the first to conceptually discuss and empirically examine the mental health strain occasioned by societal demands placed on individuals to take a COVID-19 vaccine.

## 1. Introduction

Since the onset of the COVID-19 pandemic globally, many countries have aggressively fought back to significantly curb rising infection and death rates and return their societies and economies to some semblance of normalcy. This fight typically has relied heavily on a variety of strict public health protocols, mitigation strategies, lockdown protocols and other virus containment measures [1,2]. Unfortunately, many of these measures and protocols against the spread of the virus (e.g., closures of key commercial sectors, tough and impractical physical distancing requirements, restrictions on public movement and large gatherings locally and bans on regional/international travel) have contributed to exacerbating a diverse number of social and economic issues and challenges among many populations in developed and developing countries [3,4].

With the increasing availability of highly effective COVID-19 vaccines against transmission, severe illness and associated deaths, a substantial proportion of countries have swiftly opted to procure huge stocks of vaccines to inoculate larger segments of their populations with the hope of reaching herd immunity [5]. However, a new and formidable challenge has arisen against this movement in the form of *vaccine hesitancy*, which is defined as a delay in acceptance or refusal to take a vaccine despite available vaccination services and their purported benefits [6,7]. Research claimed and cited the World Health Organization that vaccine hesitancy is a significant obstacle in the fight against the COVID-19 pandemic and, thus, remains one of the top threats affecting global health [8]. Vaccine hesitancy was found by a number of studies to be a function of myriad determinants including lack of confidence and trust in governments and public health institutions and their communication/information, excessive fear and anxiety surrounding the efficacy and side effects of the vaccines, perceived barriers, a propagation of misinformation and related conspiracies on social media on the vaccines and the virus, and even legitimate safety and health concerns among well-educated individuals [9,10,11,12]. In response, a growing number of jurisdictions have actively considered the engagement of a mandatory or forced vaccination agenda and resultant policies to overcome the sluggish uptake in vaccinations occasioned by the increasing hesitancy within their populations despite ongoing efforts to educate and build trust in the wider public space [13,14]. However, some arguments have prevailed to suggest that while mandatory vaccinations may increase vaccination coverage rates, it is unlikely to be the ‘silver bullet’ that addresses the root of the problem of hesitancy in many populations. Others have claimed that mandatory vaccination agendas and policies are likely to work in the reverse, aggravating greater reactance and resistance from anti- and pro-vaccine groups alike [14,15].

Emerging research [16] revealed that increasing pressure and demands on employees to be vaccinated resulted in lower levels of acceptance and willingness to have a COVID-19 vaccination and exacerbated vaccine concerns and distrust among surveyed health-care professionals in the UK—a phenomenon comparable to the boomerang or backfire effect. Similarly, it was acknowledged that coercive or mandatory vaccine approaches tended to worsen vaccine hesitancy due to the mediating effects of reactance—which involves negative and hostile reactions [14]. Such negative and hostile emotional and cognitive reactions can be argued to reflect or point to underlying psychological forces that might be rooted in two kinds of phenomena: psychological reactance and perceived COVID-19 vaccine pressure (or psychological/mental strain), with the latter being the central focus in the present study.

Amid the proliferation of concepts and resultant operational measures that exist to capture various forms of attitudes and perceptions of vaccinations, to the best of the authors’ knowledge, there have been no conceptual definitions and empirical accounts of *perceptions of COVID-19 vaccine pressure*. The general body of emerging literature on vaccine-related attitudes regarding COVID-19 has disclosed a variety of COVID-19 vaccine hesitancy and related constructs and measures (including attitudes towards COVID-19 vaccinations, vaccine acceptance/hesitancy, COVID-19 vaccine literacy, vaccine behavioural intentions, among others) that explore public or individual sentiment concerning decisions and behaviours associated with vaccine acceptance or refusal [17,18,19,20]. However, no study has been found that has conceptually or empirically examined individuals’ experience of anxiety, stress and/or strain as a reaction to constant and increasing societal demands on them to take a COVID-19 vaccine.

Conceptually, perceived COVID-19 vaccine pressure can be defined as internalized pressure or strain in individuals (typified as feelings of anxiety, worry, frustration, and other symptoms of mental strain) who face or experience constant or increasing societal demands and other external forms of coercion to take a [COVID-19] vaccine against which they have competing concerns or views. Existing frameworks and theories can thoroughly explain this newly conceived construct in social psychology and health psychology bodies of literature. One notable and relevant social psychological perspective that can be leveraged here is psychological reactance theory. Psychological reactance theory explains that individuals oftentimes develop and react with negative, hostile and aggressive feelings and cognitions when they believe that their behavioural freedoms are being threatened or that there is a possibility these freedoms will be restricted or eliminated [21]. Indeed, psychological reactance has been empirically shown to be activated even against well-intended persuasive educational efforts and informational campaigns to sway or change dangerous or unhealthy public attitudes on various subjects [22]. In an emerging body of research on psychological reactance to mandatory vaccination policies, it was revealed that these policies can elicit psychological reactance, in light of their restrictive or undermining effects on certain established and widely held public freedoms and choices in many democratic societies [23,24]. Moreover, this psychological reactance is intensified among people who are less supportive of mandatory vaccination policies and among those with lower vaccination intentions.

The phenomenon of *perceived COVID-19 vaccine pressure* is indeed comparable to manifestations of psychological reactance such that both constructs can be argued to be reactive in nature accompanied by similar expressions of negative and uncomfortable feelings and cognitions of aggression, stress, frustration, and anxiety. Hence, the theory of psychological reactance sets up a useful and viable reference base upon which perceived COVID-19 vaccine pressure can be conceptualised and explored. However, this paper argues that, whereas psychological reactance to vaccine pressure is rooted exclusively in the reaction to protect or preserve threatened freedoms, perceived COVID-19 vaccine pressure operates as an internalized emotive and cognitive reaction to societal pressure and demands in general that does not necessarily have to be tied to a perceived restriction of specific freedoms. Some prior studies, which utilized the Salzburg State Reactance Scale (as a measure of psychological reactance), examined participants’ negative emotions and cognitions in direct reaction to a mandatory vaccine policy and incorporated the perception that participants’ rights and freedoms were under threat of being restricted or eliminated [23,24]. Finkelstein et al. [25] also measured psychological reactance as linked to reactions of constrained freedoms and choices based on a measure developed by Hong and Page [26]. It must be reiterated that the working conceptual definition of perceived COVID-19 vaccine pressure in this study does not necessarily or explicitly attribute this experience or reaction to any specific underlying motive or reason. Therefore, although individuals who report high levels of internalized anxiety, pressure and strain in response to societal demands being placed on them to take a vaccine would subsume those who are highly sensitive and reactive to threats against their freedom to choose otherwise, not all of these individuals with heightened COVID-19 vaccine pressure react for the same reason.

In light of the aforementioned review, perceived COVID-19 vaccine pressure can best be conceptualised and understood within the context of the *stressor–strain theoretical framework*. This framework defines stressors and stain outcomes and their interrelationships in different settings and contexts and occupies a large segment of health psychology literature [27]. According to this framework, stress (or the stressor) is defined as the objective cause that has the potential to be stress-inducing based on the subjective appraisal of an individual. In contrast, strain represents the internal adverse reaction or response by the appraiser due to the excessive nature of perceived demand(s) and his/her inability to cope with the same [28]. Essentially, the notion of perceived COVID-19 vaccine pressure incorporates these assumptions by treating the individual’s appraisal of societal demands, requirements or expectations relative to vaccinations or vaccine taking as a potential stressor and the resultant internalised state of pressure (expressed as negative or unhealthy sentiments, emotions and cognitions) as the manifestation of strain. Indeed, the strain manifestations associated with perceived COVID-19 vaccine pressure are indicative of poor or adverse psychological (or mental health) reactions/symptoms of individuals who are constantly encountering or facing increased societal demands to engage in practices or behaviours (e.g., taking a COVID-19 vaccine) that are either conflicting, incompatible or excessive relative to their personal coping resources.

## 2. Research Rationale and Aims

The two key research objectives of this present study are as follows: Firstly, this study aims to conceptually introduce a new stressor–strain phenomenon or construct in the form of perceived COVID-19 vaccine pressure and an empirical operationalisation (a standardised quantitative scale/measure) of this construct. The study will provide only a preliminary or basic assessment of the psychometric properties of the scale including reliability and item analyses, interitem correlation analyses, and exploratory factor analyses (EFAs). It is expected that a more definitive psychometric assessment and validation of the instrument will take place in an upcoming ‘new sample’ of participants to fully examine its construct validity properties using confirmatory factor analyses (CFAs) and other structural equation modelling (SEM) techniques. Hence, this first objective explores an initial attempt to empirically assess and validate a newly proposed measure of this freshly conceptualised version of mental or psychological strain tied to vaccine taking demands and pressure within a given social and cultural context. The second objective of this study is to examine how this currently operationalised version of perceived COVID-19 vaccine pressure varies according to different sociodemographic factors including gender, age, education, employment status, sector of employment, monthly income, and race in this baseline sample.

There are several points of significance worthy of discussion here. One important point of significance attached to this study is its intention to make a major contribution to the existing stressor–strain framework and related literature by investigating and seeking to confirm how stressors tied to societal demands in relation to vaccine taking within a pandemic setting can potentially induce different forms of mental strain among certain categories of a population (especially among those who might be reluctant, but not necessarily opposed, to COVID-19 vaccinations).

Another potential significant contribution of the present study is that the development of a valid and reliable measure of perceived COVID-19 vaccine pressure, alongside its conceptual implications, is likely to improve the how we conceptualise and measure various forms of mental health strain associated with rising societal pressure on vaccine taking within certain sections of society. It can also help explain how vaccine hesitancy, in its various manifestations, might likely be exacerbated as an unintended (or backfired) consequence of this same pressure. Hence, it is hoped that this proposed measure of perceived COVID-19 vaccine pressure be incorporated in future empirical studies to be explored as a potential determinant (or as a mediator for other determinants) of vaccine hesitancy, vaccine taking intentions and other related behaviours in different populations.

It is important to mention that the present study was conducted in a small developing country in the English-speaking Caribbean—the nation of Barbados. At the time of writing this paper (August 2021), a large number of heated conversations, debates and public protests had taken place concerning the possible introduction of mandatory COVID-19 vaccination policies and programmes in a number of countries in the Caribbean region, including Barbados. The agenda for mandatory vaccinations has been viewed as a strategic move by some Caribbean governments by which to restart and mobilise the recovery effort in those economies that have been severely affected by the pandemic. Although a national official policy of mandatory COVID-19 vaccinations was not implemented during the time of the research process, certain groups of society had intensified their efforts to pressure, bully and coerce unvaccinated segments of the country to be inoculated or face certain consequences (e.g., using tactics such as no jab/no job policies, mandatory COVID-19 testing of unvaccinated workers on regular basis at their expense, and restrictions of certain categories of customers in certain establishments, among others). Unsurprisingly, these efforts, in turn, have been met by equally strong opposition and resistance from large segments of the masses across several Caribbean territories.

Against this backdrop, this paper offers a practical contribution amid the currently hostile and turbulent Caribbean space to expose and address the underlying dynamics and effects of constant and rising societal pressure and demands on the psychological and mental well-being of the residents of Barbados and the implications in which local and regional governments, public health authorities, employers, labour unions and mental health practitioners all play an essential role.

## 3. Methods

### 3.1. Research Context and Setting

Barbados is a small, English-speaking Caribbean country with a population of approximately 290,000, with over 90 per cent being of black African descent. The country’s main economic industry is the travel and tourism sector, which accounts for a substantial proportion (over 35%) of its gross domestic product (GDP). Indeed, this sector was the hardest hit by the COVID-19 pandemic from early 2020, resulting in a considerable number of business closures and job cuts and high levels of unemployment across the island [29]. Since the first case of COVID-19 was reported in March 2020, Barbados has recorded 4,581 cases and 48 deaths as a direct consequence of the SARS-CoV-2 virus (as of 16 August 2021). Barbados rolled out its initial national vaccination programme, with the Oxford-AstraZeneca vaccines, in early 2021 (February 2021). As of the 16 August 2021, a total of 86,141 residents have been fully vaccinated, representing approximately a third of the population [30].

### 3.2. Participants and Sampling

The target population was the adult population (18 years and over) in Barbados, which comprised approximately 229,000 residents. The minimum sample size was estimated as 384 participants based on a 95 per cent confidence level and a margin of error of 5 per cent. A total of 500 Barbadian respondents (controlling for non-response) were non-randomly targeted using an online survey distribution link via the SurveyMonkey platform. At the end of the collection process, a total of 411 useable responses were gathered. With respect to demographic profile of respondents, the majority were Black (92%), female (68%), between the ages of 25 and 55 years (65%), educated up to the tertiary level (62%), and employed full-time (61%). Over 70 per cent of respondents reported an average monthly household income of less than BDS 6000 (or less than USD 3000).

### 3.3. Instrumentation, Data-Collection and Data Analysis Procedures

A structured quantitative survey instrument comprising ten (10) Likert-scale items was designed to measure perceived (or internalised) COVID-19 vaccine pressure. On this measure, respondents were asked if they had felt, at any point of time since the start of the pandemic, a range of signs/symptoms related to psychological or mental health strain as a direct result of societal demands or pressure on them to take the COVID-19 vaccine (with scale options: 1 = Never, 2 = Rarely, 3 = Sometimes, 4 = Often, and 5 = Very Often). Sample items included: ‘Felt anxious from society threatening you to take the COVID-19 vaccine’, ‘Felt tensed from society pushing you to take the vaccine’ and ‘Felt stressed because of society’s demands on you to take the COVID-19 vaccine’. The item creation, screening and selection processes for this new measure (which were implemented prior to data collection) are discussed in the ensuing Results section of this paper. In the second part of the instrument, respondent demographics such as gender, age, employment status and sector of employment, educational background, average monthly household income and race were also captured.

During the month of August 2021, the survey was piloted, reviewed and updated and readministered to the targeted participants using an online modality (via SurveyMonkey) and an accompanying electronic link which was distributed over various social media, email and other online channels. The survey took, on average, ten minutes to complete for each respondent, and the entire data-collection process lasted for three weeks. Once all data were collated, exported and cleaned in the SPSS 27 (IBM, New York, NY, USA) programme, a range of descriptive and inferential statistics were employed and all *p*-values less than 0.05 were considered statistically significant. These are further described and discussed in the Results section.

### 3.4. Ethical Considerations

All ethical guidelines were appropriately followed based on approved standards within the local country (but no ethical board/IRB board approval was sought for this study). All participants were above the age of 18, and anonymity was assured as no personal information (such as names and other identifiers) were collected. All of the necessary ethical considerations were taken into account during the research process. All participants were duly informed about the nature and purpose of the study before they consented to participate in the survey completion process. The Survey Monkey platform notified the participants of the approximate time needed to complete the survey (12 min). The IP addresses of the participants were recorded to ensure that no participant could complete the survey more than once (ensured by the Survey Monkey response feature). Participants were assured full anonymity and confidentiality prior to their participation. Finally, all participants were told that their participation in the survey was fully voluntary and that no incentives would be provided as a result of their agreement to participate.

## 4. Results

### 4.1. Initial Scale Development Steps

International best practice recommendations in scale development processes suggest several key interrelated steps with the first two (pre-analysis) steps focusing on (a) item generation using a mixture of deductive and inductive methods and (b) theoretical content validation analysis [31,32]. Once these steps were completed, the latter phases of psychometric analyses were conducted, and the resultant results were examined.

For the first step, a review of the literature on comparable pre-existing measures and scales was conducted as a deductive method to explore relevant scale item content, structure, instructions and general wording and formatting standards. During this review process, no prior scale capturing ‘perceptions of vaccine pressure/burden/strain’ was found in the existing literature. Hence, there was a stronger reliance and focus on comparable scales covering measures of ‘psychological or mental strain’ and ‘general stress experience’ including perceived stress or strain scales (e.g., psychological strain scale—PSS and specialist doctors stress inventory—SDSI; [33,34]) and burnout scales (e.g., Copenhagen Burnout Inventory) as well as peer pressure and social conformity pressure measures (e.g., peer pressure and peer conformity scales; [35]). Accompanying this deductive method, a qualitative-based (inductive) inspection was carried out to screen selected measures and their items and a resultant pool of ten (10) Likert-scale items (ranging from 1 = never to 5 = very often) was generated.

Theoretical content validation analysis involved a review of both face and content validities of the initial scale items by three (3) qualified target population judges (or potential users of the scale) in a pilot exercise—a practice supported by Morgado et al. [32]. Although all items were approved and retained, several items were revised in terms of content and wording to reflect ‘mental health strain experience’ or internalised pressure (e.g., feeling burdened, feeling frustrated, and feeling anxious) as a result of societal pressure on COVID-19 vaccine taking. The emphasis on ‘internalised pressure’ (i.e., strain) as opposed to ‘reported levels of external societal pressure or demands’ allowed the scale to directly capture individuals’ mental health strain experience rather than their appraisal of the stressor phenomenon itself (the stressor being external societal demands/pressure). This approach deviates from a previous study [16] where ‘vaccine pressure’ was measured as an ‘external’ stressor (as opposed to a psychological strain outcome) in the form of a single-item scale capturing the level of perceived employer pressure on COVID-19 vaccinations.

Once a follow-up pre-test survey exercise was completed, the scale items and key demographic questions, manifested as a web-based or online questionnaire, were then administered to the final sample, resulting in a total of 411 useable responses. It is important to highlight that the minimum sample size requirements on developmental scale studies in the existing literature suggest a ratio of at least 10 participants per scale item (10:1) and, at best, 20 participants per scale item [32,36]. Hence, the sample size of 411 was deemed adequate for this exercise.

### 4.2. Initial Psychometric Analyses of Scale

It is important to remind the readers that the present study aimed to present initial scale development and validation results, excluding the more definitive CFA assessments which will be conducted in the second phase and discussed in an upcoming paper. It is also a common and approved methodological practice for EFA to precede CFA (in a separate sample) in scale development exercises [36]. Common factor analysis using principal axis factoring is typically best suited for theoretical and scale development purposes, over principal component analysis (PCA). For the initial psychometric assessment in the present paper, there was a reliance on principal axis factoring, interitem and item–total correlations, and reliability analyses using the Cronbach’s alpha.

Table 1 and Table 2 show the results of both principal axis factoring analysis (with Promax rotation) and the item–total correlations and related descriptive statistics for the scale items. The results of the factor analysis confirmed highly robust factorability of the data based on the significance of the Bartlett test of Sphericity (*p* < 0.001) and the high value for the KMO measure of sampling adequacy (0.96). An inspection of essential criteria (e.g., scree plot assessments, eigenvalue criterion > 1) suggested that the scale items successfully converged under a single factor, explaining 80.6% of the total variance. All factor loadings on this single factor were positive and high, ranging from 0.841 to 0.938. Further preliminary indications of good psychometric properties and unidimensionality were evidenced by all corrected item–total correlations (in Table 2) and inter-item correlations being above 0.80 (r = 0.30 is minimum standard), with an internal consistency reliability estimate of 0.97 (Table 3).

### 4.3. Perceived COVID-19 Vaccine Pressure by Respondent Demographics

Based on the second objective to explore how perceived COVID-19 vaccine pressure (as an overall scale composite) varies according to respondents’ gender, age, educational background, employment status, sector of employment, average monthly household income (in Barbados/BDS dollars), and race, a series of independent t-tests and one-way ANOVAs were requested and conducted.

Overall, there were no statistically significant differences on perceived COVID-19 vaccine pressure in relation to gender, educational background, race and monthly household income (all *p*s > 0.05). However, significant differences were revealed for age (F = 8.51, *p* < 0.001), employment status (F = 3.76, *p* < 0.01) and sector of employment (F = 2.06, *p* < 0.01). Post hoc tests were employed for the aforementioned findings to detect specific significant group differences. Firstly, respondents in the younger age groups (including 18–24, 25–34 and 35–44 age groups) reported significantly greater COVID-19 vaccine pressure than older respondents (those in the 55 years and older categories). Secondly, all categories of employed respondents (full-time and part-time employees as well as self-employed persons) experienced significantly greater vaccine pressure than retired respondents (see Table 4 for results on age and employment status). Finally, respondents employed in the tourism, hospitality and restaurant sector and those employed in government/public sector reported greater levels of vaccine pressure than unemployed respondents (see Table 5).

## 5. Discussion

The first objective of the study sought to examine initial psychometric properties of a newly proposed measure of perceived COVID-19 vaccine pressure. The initial results were indeed strong and positive, indicating a single-factor (or unidimensional) scale, with high reliability (>0.90) explaining at least 80 per cent of variance in the data. These results are consistent with established guidelines and best practice criteria for developing robust developmental scales and measures in the existing body of knowledge [31,32,36]. The findings are certainly promising concerning future conceptualisation and operationalisation work catering to the need for valid and reliable social psychological measures and tools to help us better understand the operation of various stressor–strain relationships within this pandemic environment, consistent with emerging work in this field [37,38,39].

The second objective sought to examine the demographic differences on perceived COVID-19 vaccine pressure. The results indicated that younger and employed respondents reported greater vaccine pressure than older and retired respondents in this Barbadian sample. It was also found that respondents employed in the tourism and hospitality sector and those employed in the public sector exhibited greater vaccine pressure relative to unemployed respondents. Indeed, younger and working people are much more likely to be targets of societal pressure to be vaccinated given that they represent the most economically productive groups in society and are more likely to be active in diverse public facing roles (especially, those in the tourism and government sectors). The higher mental health strain associated with vaccine pressure in this study for younger and employed respondents seems to highlight that these groups were much more psychologically affected by this societal pressure and *may* have accompanying concerns and anxieties about the vaccine itself (e.g., its safety and/or efficacy).

With respect to the age specifically, a number of studies on COVID-19 vaccine hesitancy have revealed that younger people were significantly more likely to be hesitant and even resistant to take a COVID-19 vaccine [40,41]. In terms of employment status and internalised vaccine pressure, some studies have found that vaccine hesitancy tends to be lower for the employed when compared to the unemployed [42,43] which is inconsistent with the present study’s findings. However, local context and culture must be taken into account in explaining this deviation within this Barbadian sample. It is noteworthy to understand that the private sector in Barbados, led by the Barbados Private Sector Association (BPSA), has been represented as one of the most active pressure groups behind the call for mandatory vaccinations for employees in several key sectors. The tourism and hospitality sector was the economic sector of employment placed at the centre of this debate owing to its vulnerability and volatility in this pandemic, its repeated and frequent exposure of tourism workers to international visitors to the island, and its unique contribution to national employment and economic growth [44]. At the time of data-collection (August 2021), several employers in Barbados (mostly those in the tourism, hospitality and restaurant sector) were already putting policies in place at their establishments mandating their workers to take the COVID-19 vaccine as a necessary condition for their continued employment.

Other emerging research and contemporary perspectives on mandatory COVID-19 vaccination agendas have cautioned employers against mandating or coercing employees to take the COVID-19 vaccine since this approach is more likely to exacerbate vaccine hesitancy and distrust [16,45]. In one recent study in Hong Kong which conducted a survey at two different waves (one conducted before the COVID-19 pandemic declaration and one at a later and more severe stage after the pandemic declaration), it was revealed that working individuals (especially those employed in the sales and services industries) were less willing to take the COVID-19 vaccine [46]. The underlying reasons for this low vaccine acceptance/willingness in the working population in Hong Kong were linked to health and safety concerns (e.g., possible harmful side effects) about the vaccine as opposed to those related to the efficacy of the vaccine. Therefore, it would be important to identify and understand the underlying reasons for a relatively substantial proportion of the working population of Barbados being psychologically uneasy with (or at least reluctant to) taking the COVID-19 vaccine amid increasing societal pressure to do so.

## 6. Theoretical and Practical Implications

This preliminary study and its findings have furthered our conceptual understanding of a new stressor–strain phenomenon within the existing pandemic context distinct from the well-known stressors related to the virus and its impact on public (and mental) health. This new phenomenon—*perceived COVID-19 vaccine pressure*—represents an individual’s internalised pressure or strain associated with increasing societal demands on vaccine taking relative to COVID-19. Moreover, the study contributed to the growing body of work on the operationalisation of new psychological constructs in the form of valid and reliable measures and tools for capturing psychological strain and other forms of mental health outcomes related to the pandemic (i.e., those uniquely related to vaccine taking expectations and demands).

From a practical standpoint, this newly developed tool and its application in different societies, cultures and settings can provide a range of practitioners (governments, public health officials and employers) with the necessary insights into the effects and implications of the use of coercive, mandatory or strong-arm tactics to bully or pressure certain societal groups into taking a COVID-19 vaccine against their will, personal beliefs or convictions. Such insights will allow local, regional and global authorities in certain jurisdictions like Barbados to better assess the efficacy of their own ‘overly forceful’ strategies to promote vaccine acceptance as well as provide a better understanding of other potential ‘unplanned’ drivers of vaccine hesitancy in different populations (e.g., backfire or boomerang effects). It was suggested that promoting freedom of choice for the public, building trust among people in the country, and affording avenues for regular dialogue or consultation between average citizens and their employers, public health officials, and governments on vaccine-related concerns and issues are better alternatives for improving vaccine acceptance and uptake in many societies [47].

In light of recent trends in the country and wider region where there have been increasing tensions among the labour movement, Government and private employers, it is imperative for the Government of Barbados to engage in serious discussions with trade unions, workers, employers and other key stakeholders to urgently deal with the rising societal unrest and discomfort regarding the issue of mandatory vaccinations. The authors believed that a national conversation needs to be had on the subject as well as a deeper review of the available international best practices in terms of the successful routes that different countries have taken on board to improve vaccine acceptance and uptake in their populations. One popular trend now being considered in Barbados (but not fully adopted) is the reliance of ‘safe zones’ in which only vaccinated persons will be accepted at certain types of establishments and facilities (e.g., nursing homes, hospitals, and certain types of private service environments). However, this approach is likely to be abandoned in the country due to its unpopularity among the locals and the business sector. A number of other countries have opted to forego mandatory vaccinations in favour of using ‘safe zones’ or vaccine passports. Interestingly, other countries—like the UK and Denmark (proposed in early 2022), recently have decided to lift most or all domestic COVID-19 restrictions in order to return to normal living for their citizens, even in the face of rising infections due to new variants. These countries, however, have experienced very positive responses in their vaccination and booster rollout programmes within their populations (without the reliance of coercive, unconditional mandates in place).

## 7. Limitations, Further Research Directions and Conclusions

Several limitations are worthy of mention here. Firstly, the study adopted a cross-sectional survey research design that does not allow for causal inferences among different variables correlated with perceived vaccine pressure and precludes temporal observations of the scale’s predictive validity (over time). Longitudinal research will offer this benefit in future scale development exercises. The use of self-report measures, alongside a convenience sampling method and a web-based survey modality, raises the prospect of participant selection bias, social desirability bias, response sets, and coverage and nonresponse biases. Finally, the study did not rely on other stronger assessments of psychometric validation to assess the scale’s construct validity (e.g., convergent and discriminatory validities) based on CFA and more sophisticated techniques. Further research is expected to conduct more rigorous validations of this new proposed measure in different samples and cultures. It is also recommended that future research studies incorporate this measure of perceived COVID-19 vaccine pressure to explain variations in vaccine hesitancy and other related attitudes and behaviours associated with vaccine taking as well as variations in other mental and physical health outcomes of individuals (e.g., exhaustion/burnout, depression, anxiety, and physical health status). It would also not be surprising for this new scale to be adapted to measure internalised pressure or strain associated with other types of vaccine-taking demands and scenarios (e.g., the societal demands on influenza vaccine taking). Finally, it is important that the concept of perceived vaccine pressure be elevated as a critical mental or psychological strain phenomenon that is comparable to other types of mental health constructs and variables in the wider body of the existing literature, especially within the COVID-19 pandemic era.

## Figures and Tables

**Table 1:** EFA results.

Scale Items	Factor 1
Frustrated from society pestering you to take the COVID-19 vaccine.	0.875
Burdened from society forcing you to take the COVID-19 vaccine.	0.918
Anxious from society threatening you to take the COVID-19 vaccine.	0.902
Tensed from society pushing you to take the COVID-19 vaccine.	0.930
Stressed because of society’s demands on you to take the COVID-19 vaccine.	0.938
Attacked from society’s insistence that you take the COVID-19 vaccine.	0.881
Overwhelmed from societal pressure on you to take the COVID-19 vaccine.	0.934
Afraid because society is bullying you to take the COVID-19 vaccine.	0.851
Pressured from society demanding you to take the COVID-19 vaccine.	0.907
Deeply troubled because society is mandating that you take the COVID-19 vaccine.	0.841

Extraction Method: Principal Axis Factoring with Promax Rotation.

**Table 2:** Corrected item–total correlations.

Scale Items	Mean	SD	Corrected Item-Total Correlation
Frustrated from society pestering you to take the COVID-19 vaccine.	2.451	1.512	0.864
Burdened from society forcing you to take the COVID-19 vaccine.	2.208	1.433	0.905
Anxious from society threatening you to take the COVID-19 vaccine.	2.109	1.420	0.889
Tensed from society pushing you to take the COVID-19 vaccine.	2.183	1.435	0.917
Stressed because of society’s demands on you to take the COVID-19 vaccine.	2.148	1.448	0.925
Attacked from society’s insistence that you take the COVID-19 vaccine.	2.074	1.441	0.870
Overwhelmed from societal pressure on you to take the COVID-19 vaccine.	2.089	1.439	0.921
Afraid because society is bullying you to take the COVID-19 vaccine.	1.903	1.309	0.840
Pressured from society demanding you to take the COVID-19 vaccine.	2.176	1.473	0.896
Deeply troubled because society is mandating that you take the COVID-19 vaccine.	2.367	1.587	0.831
OVERALL SCALE MEAN (1 to 5)	2.170	1.321	-

Note: Scale ranged from 1 (Never) to 5 (Very Often).

**Table 3:** Reliability estimate and average inter-item correlation statistics.

	Mean	Minimum	Maximum	N of Items
Inter-Item Correlations	0.806	0.700	0.890	10

Cronbach’s Alpha = 0.97.

**Table 4:** Perceived COVID-19 vaccine pressure by age and employment status.

**Age**	**Mean**	**SD**	**F-Statistic**	***p***
*18–24	**2.39**	**1.18**	8.51	0.000 ***
*25–34	**2.38**	**1.34**		
*35–44	**2.64**	**1.51**		
45–54	2.17	1.38		
*55–64	**1.74**	**0.98**		
*65+	**1.34**	**0.66**		
**Employment Status**	**Mean**	**SD**	**F-statistic**	***p***
*Employed, working full-time	**2.28**	**1.37**	3.76	0.001 **
*Employed, working part-time	**2.56**	**1.37**		
*Self-Employed	**2.40**	**1.38**		
Not employed, looking for work	2.19	1.21		
Not employed, NOT looking for work	1.77	1.03		
*Retired	**1.45**	**0.77**		
Disabled, not able to work	1.71	1.46		

Note: *** *p* < 0.001, ** *p* < 0.01. Scale scores ranged from 1 to 5. Bold-faced categories with asterisks were involved in significant differences.

**Table 5:** Perceived COVID-19 vaccine pressure by sector of employment (employed respondents only).

Sector of Employment	Mean	SD	F-Statistic	*p*
Advertising and Marketing	1.90	1.55	2.06	0.005 **
Agriculture	1.57	0.45		
Airlines and Aerospace (including Defence)	1.00	0		
Automotive	1.97	0.93		
Business/Management Support Services	2.18	1.27		
Construction	2.43	1.41		
Education	2.24	1.43		
Entertainment and Leisure	3.60	1.54		
Finance, Insurance and Financial Services	1.88	1.36		
*Tourism, Hospitality and Restaurants	**2.60**	**1.32**		
*Government	**2.45**	**1.39**		
Healthcare and Pharmaceuticals	2.01	1.30		
Manufacturing	2.68	0.95		
Nonprofit	3.55	0.63		
Personal care services (e.g., barber, hairdresser, salon worker)	2.32	1.44		
Retail and Wholesale	2.26	1.28		
Real Estate	2.37	1.22		
Telecommunications, Technology, Internet and Electronics	1.73	1.384		
Transportation and Delivery	1.40	0.56		
Utilities, Energy, and Extraction	2.28	1.41		
Other Services	2.42	1.46		
*Currently Not Employed	**1.46**	**0.72**		

Note: ** *p* < 0.01. Scale scores ranged from 1 to 5. Bold-faced categories with asterisks were involved in significant differences.

## Data Availability

The datasets generated during and/or analysed during the current study are available from the corresponding author on reasonable request.
